# Plant Nutrition: Physiological and Metabolic Responses, Molecular Mechanisms and Chromatin Modifications

**DOI:** 10.3390/ijms23084084

**Published:** 2022-04-07

**Authors:** Fatema Binte Hafiz, Sabine von Tucher, Wilfried Rozhon

**Affiliations:** 1 Department of Agriculture, Ecotrophology and Landscape Development, Anhalt University of Applied Sciences, 06406 Bernburg, Germany; fatema.hafiz@hs-anhalt.de; 2 TUM School of Life Sciences, Technical University of Munich, 85354 Freising, Germany; sabine.tucher@tum.de

Plant growth and crop yield highly depend on the availability of all required nutrients, ideally in well-balanced ratios. However, plants often face shortages of one or several nutrients. Plant hormones play an important role in the adaptation of plant growth during nutrient-deficiency stresses. Therefore, the aim of this Special Issue is to elucidate the impact of nutrients on plant productivity, root and shoot morphology, as well as to determine the underlying mechanisms involved in nutrient deficiency responses. The manuscripts submitted for this Special Issue contribute to unravelling the molecular mechanisms involved in the regulation of phenotypic and metabolic responses to nutrient limitations, particularly in the case of nitrogen (N), phosphate (P_i_) and iron (Fe) deficiency. Systemic nutrient deficiency responses were extensively studied by transcriptomic, proteomic and metabolomic analyses in the economically important crop plants rice and maize and in the model plant *Arabidopsis thaliana*.

Phosphorus is an important macronutrient for plant development. It is a constituent of numerus biomolecules including nucleic acids, phospholipids, co-substrates and co-factors, for instance, adenosine triphosphate (ATP), nicotinamide adenine dinucleotide phosphate (NADPH) and pyridoxal phosphate. Phosphate plays a vital role in plant metabolic functions, including photosynthesis, respiration [1] as well as in signal transduction [2,3,4]. Depending on the pH, plants take up inorganic phosphorus from soil in the form of dihydrogen phosphate, H_2_PO_4_^−^, and hydrogen phosphate, HPO_4_^2−^. P_i_ deficiency responses, transduced by root P_i_ transporters and phytohormones, can cause diverse phenotypic, transcriptional and metabolic alterations in different plant species. For example, in *Arabidopsis*, P_i_ deficiency markedly reduces primary root elongation by inhibiting meristematic cell elongation and proliferation and increases the root-to-shoot ratio due to a stronger decrease in shoot compared to root growth [5,6]. Auxin can modulate the root morphology in response to P_i_ supply. In P_i_ deficiency, auxin levels increase in plants and enhance lateral root formation but reduce primary root growth.

Sun et al., 2019 [7] elucidated that the auxin-efflux carrier protein PIN2 regulates basipetal auxin transport in roots and plays a key role in root growth and development under P_i_ deficiency in *OsPIN2*-overexpressing rice. The *OsPIN2* overexpression line also showed increased auxin distribution in roots, which increased root hair development in rice.

Chutia et al., 2021 [8] showed the relationship between P_i_ and Fe deficiency signals at the phenotypic and metabolic levels. Fe deficiency causes chlorosis, characterized by chlorophyll loss and subsequent yellowing of leaves [9], while P_i_ shortage leads to stunted shoots and dark green leaves [10]. The authors extensively analyzed the metabolic responses of *A. thaliana* shoots and roots under single and combined P_i_ and Fe deficiencies. Reduced root, shoot and seedling growth was recorded in response to P_i_ scarcity while Fe deficiency enhanced primary metabolite formation such as asparagine, leucine and tyrosine. Both P_i_ and Fe deficiency-responsive transcription factors and metabolic regulators controlled the metabolic changes. Notably, organic acid (malate and citrate) formation increased in roots in response to combined P_i_ and Fe deficiencies. Malate and citrate can solubilize iron and thereby enhance its uptake [11], a function similar to that of hydroxycoumarines, which were recently reported to act as chelators to mediate iron-uptake by plants [12,13,14,15]. 

Similar to P_i_, N is also an important macronutrient required for plant growth and development. N deficiency can adversely affect photosynthesis, inhibit leaf area growth and eventually reduce the longevity of leaves [16]. Inorganic N is taken up by plant roots in the form of nitrate (NO_3_^−^) and ammonium (NH_4_^+^) [17] and transported to the upper plant bodies by several NO_3_^−^ transporters (NRTs) [18,19]. In this context, Ma et al., 2020 [20] elucidated that the high-affinity transporter NRT3.1 forms a complex with NRT2.5 on the plasma membrane of root hairs, which, in turn, increased NO_3_^−^ and NH_4_^+^ uptake and enhanced the growth of *A. thaliana* seedlings under N starvation.

In a very interesting manuscript, Wang et al., 2020 [21] investigated the impact of gibberellins (GAs) in nitrogen uptake. Low N supply suppressed the production of bioactive GAs, particularly GA_1_. Experiments with ^15^N-labelled nitrate revealed significantly reduced nitrate uptake by the *zmga3ox* mutant, a maize line with reduced GA biosynthesis, both under low and high N supply. Treatment with the gibberellin GA_3_ could rescue the mutant to some extent, while the application of uniconazole, a potent triazole-type GA biosynthesis inhibitor, reduced ^15^N-nitrate uptake in the wildtype line. RNA sequencing and qPCR analysis showed that GAs modulate the transcript abundance of nitrate uptake-related genes, including the high affinity nitrate transporters *ZmNRT2.1* and *ZmNTR2.2*, as well as the nitrate transporter 1/peptide transporters *ZmNPF6.3a* and *ZmNPF6.3b*. In addition, a number of transcription factors known to be involved in expression regulation of nitrate transporters also showed different mRNA abundances in wild-type and *zmga3ox* plants cultivated under low and high nitrate conditions. These experiments provide convincing evidence that GAs are crucial for efficient upregulation of nitrate transporter abundance and therefore for high nitrogen use efficiency (NUE). With respect to that, it is important to mention that a number of high-yield varieties of cereal crops that enabled the “green revolution” are weak GA biosynthesis or signaling mutants [22]. While weak GA deficiency causes semi-dwarfism and therefore increases the yield, the same mechanism reduces NUE. Under conditions with high fertilization, this is of minor importance. However, under low fertilization, as it is required for limiting emission of the strong greenhouse gas N_2_O, the performance of such lines might be strongly affected. Clearly, more research is needed to investigate the impact of these findings under field conditions.

Liang et al., 2021 [23] performed phenotypic, transcriptomic and proteomic analyses on roots of a rice variety with high nitrogen use efficiency grown hydroponically in the presence of different levels of ammonium nitrate. The authors observed that root length was highest at low levels of ammonium nitrate, while it was significantly lower in the presence of no as well as high levels of ammonium nitrate. Proteomic analysis revealed that glutamine synthetase (OsGS2) was upregulated 48-fold at low ammonium nitrate levels. In addition, the GS activity in roots increased at low N supply, although only less than twofold and thus to a much lower level than the OsGS2 protein abundance. In addition, the abundance of glycolytic enzymes was affected in a way that 2-phosphoenolpyruvate (PEP) synthesis may be activated under low N supply conditions. The entrance of PEP into the citrate cycle may stimulate the production of 2-oxoglutarate (2-OG), which serves as a precursor for the synthesis of glutamate and glutamine. Recently, glutamine was shown to act, in addition to its role in metabolism, as a signaling molecule to induce the expression of key transcription factor genes involved in nitrogen and stress responses in rice roots [24]. Based on these results, the authors proposed an alternative pathway of N assimilation in response to N deficiency. However, although the proteomic results speak for this hypothesis, it will be important to substantiate this idea by analysis of metabolite levels and fluxes (particularly of 2-OG) and quantification of enzymatic activities. As expected, the authors also found significant increases in the abundances of nitrate transporters (NRTs), nitrate reductase (NR2) and ammonium transporters (AMTs). Furthermore, the data provide evidence for modulation of the phenylpropanoid pathway in response to N-deficiency stress.

Taken together, the data presented in this Special Issue elucidate that plants undergo complex molecular processes and metabolic changes to adjust their growth under P_i_-, Fe- and N-deficient stresses (Figure 1).

## Figures and Tables

**Figure 1:**
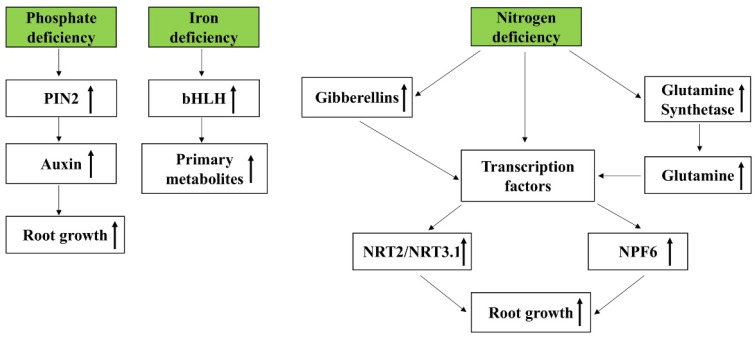
Schematic representation of molecular mechanisms involved in response to Pi, Fe and N deficiencies. PIN2, pin-formed 2, a component of the auxin efflux carrier; bHLH, basic helix–loop–helix transcription factors; NRT2, nitrate transporter 2; NRT3.1, nitrate transporter 3.1; NPF6, nitrate transporters of the nitrate transporter 1/peptide transporter class.
